# Editorial: Online cognitive behavioral therapy for insomnia

**DOI:** 10.3389/frsle.2025.1557003

**Published:** 2025-03-10

**Authors:** Stuart F. Quan

**Affiliations:** ^1^Division of Sleep and Circadian Disorders, Brigham and Women's Hospital, Harvard Medical School, Boston, MA, United States; ^2^Division of Pulmonary, Allergy, Critical Care and Sleep Medicine, University of Arizona College of Medicine, Tucson, AZ, United States

**Keywords:** insomnia, cognitive behavioral therapy, internet, digital, online

Chronic insomnia disorder afflicts ~10% of adults. Affected persons often suffer from a variety of mental health issues including anxiety, depression, and substance abuse. Impairment in concentration, memory, and decision making also occurs. Moreover, chronic insomnia is associated with an increased risk of a variety of physical health conditions such as cardiovascular disease, diabetes and obesity as well as a reduction lifespan (Roth, 2007).

Cognitive behavioral therapy or CBT-i is the preferred treatment for chronic insomnia (Qaseem et al., 2016). However, traditional CBT-i requires in-person interaction by a clinician trained in Behavioral Sleep Medicine. Unfortunately, there are relatively few trained therapists in comparison to the number of persons with chronic insomnia (Thomas et al., 2016). Increasingly, digital or online programs are being developed to increase the availability of CBT-i. Although these programs generally are consistent with the concepts used in traditional CBT-i, in many cases their effectiveness is not yet demonstrated. In this Research Topic, four articles are included which address insomnia outcomes after online digital CBT-i (dCBT-i) or the latter's impact when there is co-morbid sleep apnea and shift work.

Although CBT-i is the initially recommended treatment for chronic insomnia, remission of insomnia occurs in only two-thirds of patients (Jernelöv et al., 2022). Identification of patients who are less likely to respond is important in the shared decision making that is recommended in selection of a treatment option (Qaseem et al., 2016). If it can be determined that response to a digital or online program is unlikely, an alternative therapy with a better chance for treatment success may be offered. The article by Pchelina and Poluektov offer insights into who is most likely to improve with an internet-based dCBT-i program. They found that the most important predictors of treatment success were shorter duration of insomnia and better attitude about expected treatment success. In addition, more successful outcomes were observed in those with lower levels of attention seeking, grandiosity, distractibility, and rigid perfectionism.

It is now recognized that the prevalence of insomnia symptoms among patients with obstructive sleep apnea may be as high as 58%, a condition commonly referred to as COMISA (Luyster et al., 2010). Although CBT-i is the recommended initial therapy for chronic insomnia, there is concern that sleep restriction which is used as part of CBT-i may exacerbate the daytime somnolence in those with obstructive sleep apnea. This may be of particular concern if the CBT-i is not administered directly by a trained therapist. In the study by Sweetman et al., the impact of an interactive dCBT-i program was evaluated in study participants with insomnia alone and those with suspected COMISA. Both groups had improvement in their insomnia symptoms. Importantly, there was no worsening of sleepiness, thus allaying concerns that dCBT-i may increase somnolence in some patients with COMISA.

It is not always appreciated that insomnia occurs in children and adolescents. However, in comparison to adults, research related to treatment of insomnia in children is relatively limited, especially programs that do not involve in-person interaction. The review by Lah and Cao is a valuable summary of the small number of studies in which behavioral interventions for insomnia were remotely delivered. They found that remote interventions were generally comparable to in-person therapy with improvements in most sleep parameters and mood. However, only seven studies were included in their review which underscores the need for additional research.

Chronic insomnia is a frequent manifestation of shift workers. Although the insomnia is produced by circadian misalignment resulting from irregular work hours or night work, CBT-i can be effective in managing insomnia symptoms in these patients. Tout et al. review factors responsible for sleep problems including insomnia in shift workers and make treatment recommendations including the use of online dCBT-i programs. They emphasize that such programs may need modifications to accommodate shift workers who already may have restricted sleep and by necessity work irregular hours. However, some studies have shown benefit.

Chronic insomnia is a highly prevalent condition in our society. It adversely affects mental and physical health and performance (Roth, 2007). Although CBT-i is the preferred treatment and is effective, there is a shortage of qualified clinicians to provide in-person therapy (Thomas et al., 2016). With the ubiquity of the internet and increasing acceptance of its use for healthcare purposes, online or digital delivery methods have the potential of increasing access to CBT-i. The articles in this Research Topic highlight the promise of online dCBT-i. However, before widespread adoption, additional research is needed to identify the patient population for whom dCBT-i is beneficial and the best approaches for delivery. In addition, further comparative effectiveness trials of digital or online vs. clinician delivery and hypnotic medications should be performed.
